# Spontaneous Intracranial Hemorrhage Associated With an Intracranial Meningioma

**DOI:** 10.7759/cureus.40472

**Published:** 2023-06-15

**Authors:** Bill R Ferrufino-Mejia, Héctor A Rodríguez-Rubio, Rodrigo López-Rodríguez, Alfredo Bonilla Suastegui, Marco A Rodríguez-Florido, Flavio Hernandez-Gonzalez, Alan Ferrufino-Mejia

**Affiliations:** 1 Neurosurgery, National Institute of Neurology and Neurosurgery Manuel Velasco Suárez, Mexico City, MEX; 2 Neurosurgery, Mexican Institute of Social Security (IMSS) XXI Century National Medical Center, Mexico City, MEX; 3 Pathology, Mexican Institute of Social Security (IMSS) XXI Century National Medical Center, Mexico City, MEX; 4 Neurosurgery, Hospital Juárez de México, Mexico City, MEX

**Keywords:** intracranial hemorrhage, intratumoral hemorrhage, neurosurgical treatment, subdural hematoma, meningioma

## Abstract

Spontaneous intracranial hemorrhage associated with an intracranial meningioma is rare, with a reported incidence of below 2.4% of all meningiomas. Such cases are described with a cause subdural with intratumoral hemorrhage, which is a challenge for patients and healthcare professionals because it can occur spontaneously without other pathological antecedents. We describe the case of a 55-year-old woman with subdural hemorrhage over the frontoparietal region of the right hemisphere associated with a meningioma, generating a mass effect and shifting the third ventricle and lateral ventricle. Therefore, urgent surgical treatment was decided. A tumor lesion was found with apoplexy, soft consistency, and violaceous color with abundant vascularity in the lesion’s center, suggesting a probable angiomatous meningioma. The histopathological evaluation confirmed meningothelial hemorrhagic meningioma grade I, according to the World Health Organization grading. This article discusses the causes, risk factors, diagnosis, and surgical treatment for hemorrhage associated with intracranial meningioma.

## Introduction

Although most meningiomas are benign and slow-growing, they can occasionally present with significant complications, such as hemorrhage. Hemorrhage associated with intracranial meningioma poses unique challenges for patients and healthcare professionals [1]. In cases described in the literature, about 0.5-2.4% of all meningiomas are estimated to present with intracranial hemorrhage [2]. This article discusses the causes, diagnosis, and treatment options for hemorrhage associated with intracranial meningioma.

## Case presentation

We present the case of a 55-year-old woman with a history of peripheral venous disease, arterial hypertension, and diabetes mellitus. Her illness began upon awakening with a sudden oppressive onset of severe holocranial headache which intensified and was accompanied by nausea and vomiting of gastric contents, paresthesia in all four extremities, and subsequent loss of alertness. She was admitted to the emergency room with a Glasgow Coma Scale score of 7 (E2, V1, M4) without neurological focalization.

**Figure 1:**
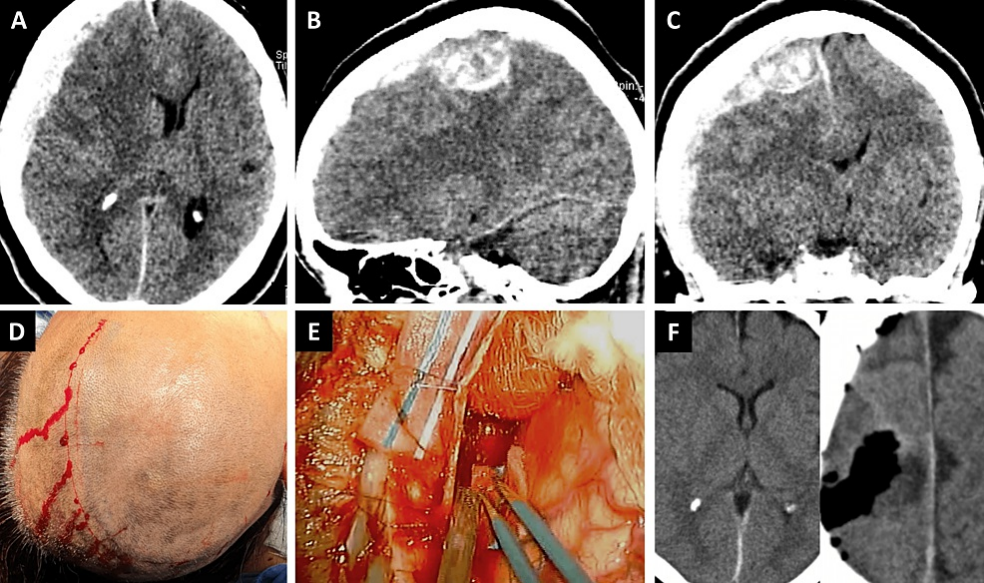
CT studies and view of the surgical approach. A preoperative CT study (axial view) demonstrates a right-sided subdural hemorrhage over the frontal lobe (A) and over the parietal lobe (sagittal view) (B). A midline shift is accompanied by the collapse and compression of the third and right lateral ventricles (C). Falconer’s approach was performed with subsequent frontoparietal-temporal craniotomy (D). The hematoma was drained, and the tumor was excised (E). Postoperative CT demonstrates reversal of the midline shift and meningioma resection (F).

CT revealed extra-axial hemorrhage measuring 13 × 14 × 9 mm which was located over the frontoparietal region of the right hemisphere, was associated with a tumor, generating a mass effect and displacing the midline by 14 mm, and was accompanied by compression of the third ventricle and right lateral ventricle, for which urgent surgical treatment was decided (Figure 1).

**Figure 2:**
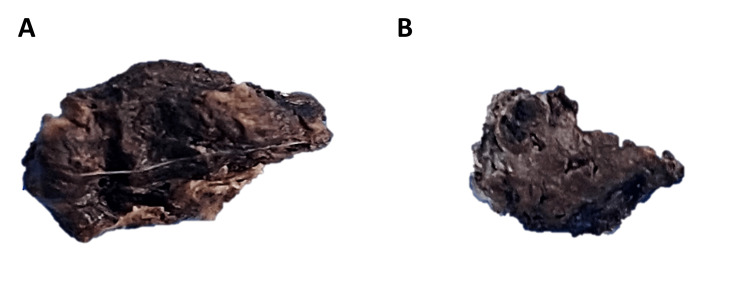
Macroscopic study of the surgical specimen. Rough specimen with a dark brown anfractuous external surface with a congestive aspect (A). Cut surface with hemorrhagic aspect (B).

Right, Falconer’s approach was performed with subsequent frontoparietal-temporal craniotomy. After a C-shaped dural opening with subsequent hematoma drainage, a tumor lesion was found with apoplexy, sustainable consistency, and violaceous color with abundant vascularity in the center of the lesion, suggesting a probable angiomatous meningioma (Figure 2). The definitive anatomic pathology report was an irregular specimen measuring 1.5 × 1 × 1 cm with a dark brown, congestive, anfractuous external surface (Figure 2A). The lesion was solid when cut with a hemorrhagic aspect (Figure 2B).

**Figure 3:**
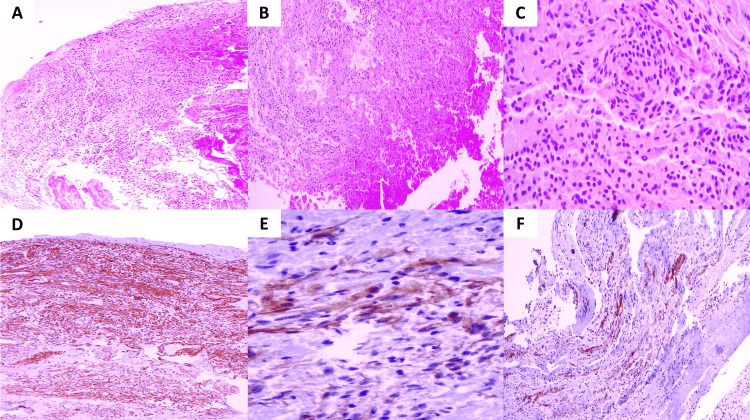
Photomicrographs of the surgical specimen, hemorrhagic meningothelial meningioma. Panoramic view of the neoplastic lesion with hemorrhagic areas, HE 4× (A and B). Neoplastic meningothelial cells, HE 40× (C). Diffuse expression of vimentin, IHC 4×. (D) Focal expression of EMA, IHC 40× (E). Patchy expression of SSTR2A (F). HE: hematoxylin and eosin; IHC: immunohistochemistry; EMA: epithelial membrane antigen; SSTR2A: somatostatin receptor 2a

The histopathological evaluation confirmed a neoplastic meningeal lesion organized in meningothelial nests with hemorrhagic areas. On immunohistochemistry, the lesion expressed vimentin with a diffuse pattern, focal areas of epithelial membrane antigen expression, and patches of immunolabeling with somatostatin receptor 2a. A diagnosis of hemorrhagic meningothelial meningioma was confirmed (Figure 3).

The neurological status improved markedly with a Glasgow Coma Scale score of 14, along with a gradual improvement in neurological data. The patient was discharged seven days after the surgery.

## Discussion

Intracranial hemorrhage in patients with meningiomas is a rare event. In general, meningiomas are slow growing and highly vascularized. Some studies have reported a hemorrhage rate of approximately 0.5% to 2.4% of all patients with meningiomas [1,2]. Attempts have been made to describe the pathophysiological mechanisms of hemorrhage. Some mechanisms proposed in the literature are related to the characteristics of the tumor vasculature due to rapid angiogenesis resulting in vessels with thinned, friable vascular walls, associated with direct vascular invasion by tumor cells like stretching subdural veins [3-6]. These characteristics predispose to an increased risk of bleeding due to minor trauma, hypertension, blood dyscrasias, and anticoagulation. The tumor histological subtype (except for angioblastic histology), age, and sex of the patient do not demonstrate a significant relationship with the hemorrhagic event [3]. Bošnjak et al. (2005) proposed age over 70 years; convexity or ventricular location; malignant, fibrous, or angioblastic histology of the tumor; history of hypertension; anticoagulation therapy; and traumatic brain injury as risk factors for hemorrhage in patients with meningiomas also influenced the clinicopathological features of patients with intracranial bleeding from unsuspected meningioma. They related these data to the surgery-related outcome. The study demonstrated that meningotheliomatous meningiomas were not associated with an increased propensity to bleed [1]. The possibilities related to the pathophysiology of these bleeding events include bleeding within the tumor (intratumoral hemorrhage) or on its surface (intracerebral hemorrhage, subdural hematoma, and subarachnoid hemorrhage) presenting as events with rapid neurological deterioration and increased intracranial pressure [7]. Diagnostic imaging is performed by CT which is associated with high sensitivity for intracranial hemorrhages. The study of choice for brain tumors is MRI. In some cases, cerebral angiography may be performed to evaluate the blood supply to the tumor and identify any abnormal vascular structures, which aids in surgical planning [8]. However, a conservative approach with close surveillance may be considered in some instances, particularly with small and asymptomatic hemorrhages [9]. On the other hand, surgical resection is the primary treatment modality for meningiomas and is usually recommended for symptomatic tumors or those causing a mass effect; however in cases where surgical intervention carries higher risks or is not feasible, stereotactic radiosurgery may be considered [10].

## Conclusions

Spontaneous hemorrhage associated with meningioma is rare, and the pathophysiological mechanisms, etiology, and risk factors remain unclear. The variety of neurological states depends on each patient and the location of the lesion. Surgical resolution of hemorrhagic meningioma and hematoma in a single event is the treatment of choice. The prognosis is good and depends on the tumor characterization and the associated tumor malignancy.
